# Integrating single-cell analysis and machine learning to create glycosylation-based gene signature for prognostic prediction of uveal melanoma

**DOI:** 10.3389/fendo.2023.1163046

**Published:** 2023-03-23

**Authors:** Jianlan Liu, Pengpeng Zhang, Fang Yang, Keyu Jiang, Shiyi Sun, Zhijia Xia, Gang Yao, Jian Tang

**Affiliations:** ^1^ Department of Plastic and Burns Surgery, The First Affiliated Hospital of Nanjing Medical University, Nanjing, China; ^2^ Department of Thoracic Surgery, The First Affiliated Hospital of Nanjing Medical University, Nanjing, China; ^3^ Department of Ophthalmology, Charité – Universitätsmedizin Berlin, Campus Virchow-Klinikum, Berlin, Germany; ^4^ Department of General, Visceral, and Transplant Surgery, Ludwig-Maximilians-University Munich, Munich, Germany

**Keywords:** glycosylation, uveal melanoma, immunotherapy, machine learning, AUP1

## Abstract

**Background:**

Increasing evidence suggests a correlation between glycosylation and the onset of cancer. However, the clinical relevance of glycosylation-related genes (GRGs) in uveal melanoma (UM) is yet to be fully understood. This study aimed to shed light on the impact of GRGs on UM prognosis.

**Methods:**

To identify the most influential genes in UM, we employed the AUCell and WGCNA algorithms. The GRGs signature was established by integrating bulk RNA-seq and scRNA-seq data. UM patients were separated into two groups based on their risk scores, the GCNS_low and GCNS_high groups, and the differences in clinicopathological correlation, functional enrichment, immune response, mutational burden, and immunotherapy between the two groups were examined. The role of the critical gene AUP1 in UM was validated through *in vitro* and *in vivo* experiments.

**Results:**

The GRGs signature was comprised of AUP1, HNMT, PARP8, ARC, ALG5, AKAP13, and ISG20. The GCNS was a significant prognostic factor for UM, and high GCNS correlated with poorer outcomes. Patients with high GCNS displayed heightened immune-related characteristics, such as immune cell infiltration and immune scores. *In vitro* experiments showed that the knockdown of AUP1 led to a drastic reduction in the viability, proliferation, and invasion capability of UM cells.

**Conclusion:**

Our gene signature provides an independent predictor of UM patient survival and represents a starting point for further investigation of GRGs in UM. It offers a novel perspective on the clinical diagnosis and treatment of UM.

## 1 Introduction

Uveal melanoma (UM), the most common type of intraocular cancer in adults, originates from melanocytes in the uvea, which includes the iris, ciliary body, and choroid (1). UM accounts for 3% to 5% of all melanoma and 79% to 81% of ocular melanoma (2). The global average incidence of UM ranges from 0.002‰ to 0.008‰, with significant geographic and ethnic disparities (3). Around 50% of UM patients experience hematogenous metastasis, with the liver being the primary and most common site of metastasis (4, 5). Several studies have been conducted to prevent metastasis in UM, high-dose interferon is the only adjuvant therapy shown to improve recurrence-free survival time and control the primary UM. However, there has been no significant improvement in overall survival (OS) or metastasis-free survival in any of these studies (1).

Patients with metastatic UM have a median survival time of 6 to 12 months, and their prognosis heavily depends on liver metastasis and disease progression in the liver (6). Despite the numerous studies by scholars exploring various immunotherapies, such as immune checkpoint inhibitors (ICI), cancer vaccines, and T-cell passaged cell therapy (7, 8), the effects of immunotherapy for UM have been disappointing (9). Given the limited therapeutic options for UM, it is crucial to investigate its underlying pathophysiological pathways and develop a reliable prognostic prediction model for UM patients.

Glycosylation is a biological process that occurs through the action of various glycosyltransferases (GTs) and glycosidases (10, 11). This modification changes the protein’s conformation and structure, which in turn affects its functional activity (12). The regulation of glycosylation is controlled by glycogenes, which are genes that encode for glycosidases and sulfotransferases. An abnormal expression or regulation of these genes is linked to tumor progression and is considered a hallmark of cancer (13, 14). A translational study showed that the expression levels of 210 GTs genes could differentiate between six types of cancer, including breast cancer and ovarian cancer. Moreover, glycosylation has the potential to act as a prognostic indicator, as a signature of glycosylation-related genes (GRGs) was shown to predict clinical outcomes in ovarian cancer patients (15). Other post-translational regulatory mechanisms, such as ubiquitination (16), phosphorylation (17) and epigenetic modifications (18) have also been reported as potential biomarkers in UM prognostic models. However, despite being one of the most crucial post-transcriptional alterations among the 300 protein modifications, few studies have explored the relationship between GRGs signature and the tumor microenvironment (TME) of UM.

To address this gap, we leveraged bulk RNA-seq and scRNA-seq data to establish the GRGs signature in UM and divided UM patients into GCNS_low and GCNS_high groups using a selected cut-off value. Our analysis revealed a significant difference in prognosis between the two groups. The results were validated using the GSE84976 dataset from the Gene Expression Omnibus (GEO) database. Furthermore, we examined the utility of the GRGs signature in the TME, tumor mutational burden (TMB), immunotherapy response, and drug sensitivity. Lastly, we explored the impact of inhibiting AUP1 expression on UM cell proliferation and migration *in vitro*. Our study provides novel insights into the role of glycosylation in UM and holds promise for improved patient stratification and targeted therapy development.

## 2 Materials and methods

### 2.1 Data acquisition

The scRNA-seq data of UM was obtained from the GEO (https://www.ncbi.nlm.nih.gov/geo/), which comprised 59,915 tumor and non-tumorous cells from eight primary and three metastatic samples (accession number: GSE139829). The RNA expression profiles, gene mutations, and relevant clinical information of UM were extracted from The Cancer Genome Atlas (TCGA) database (https://tcgadata.nci.nih.gov/), with a sample size of 80 and served as the training dataset. The FPKM format of the TCGA-UM was transformed into the TPM format. Additionally, the expression profiles of GSE84976 were obtained from the GEO database and used as the validation set. Before any further analysis, all transcriptome data were log2-transformed. The “sva” package adjusted for batch effects between TCGA-UM and GSE84976. The GeneCards database (https://www.genecards.org/) was consulted to obtain GRGs, and a total of 110 GRGs with a relevance score greater than 1.0 were selected for further analysis. To assess the prognostic utility of the risk score in ICI therapy, we utilized the IMvigor 210 Core Biologies database of patients with advanced urothelial cancer undergoing anti-PD-L1 immunotherapy, which was analyzed using the R program (19).

### 2.2 Data processing and annotation

We employed the “seurat” and “singleR” R packages to perform quality control on scRNA-seq data (20). To ensure the data’s accuracy, we eliminated genes expressed in less than three single cells, cells with less than 200 or more than 7,000 genes, and cells with more than 10% mitochondrial genes. Out of the total, 30,934 cells were selected for further analysis. These cells underwent scaling after normalization through a linear regression model that utilized the log-normalization method. Using the “FindVariableFeatures” function, we identified the top 3,000 hypervariable genes. To remove batch effects that may affect downstream analysis, we utilized the “FindIntegrationAnchors” function of the canonical correlation analysis (CCA) method. We integrated and scaled the data using the “IntegrateData” and “ScaleData” functions, determined the anchor points by principal component analysis (PCA), and evaluated the top 20 PCs using the t-distributed stochastic neighbor embedding (t-SNE) algorithm to discover significant clusters. We used the “FindNeighbors” and “FindClusters” functions (resolution =0.8) to obtain 24 cell clusters, which were visualized as a t-SNE diagram. The “FindAllMarkers” function in the “seurat” package was applied to identify the differentially expressed genes (DEGs) in each cluster. The “singleR” package annotated cell types based on the cluster’s canonical marker genes, which were later manually validated against published literature (21).

### 2.3 AUCcell

The “AUCell” R package was utilized to determine the active status of gene sets in scRNA-data by assigning glycosylation activity scores to each cell lineage (22). The gene expression rankings of each cell were calculated based on the AUC value of selected GRGs to assess the fraction of highly expressed gene sets. Cells with larger AUC values had higher gene expression levels. The “AUCell_exploreThresholds” function was used to identify cells actively involved in glycosylation gene sets. These cells were then grouped into high and low G-AUC categories using AUC score cutoff values and visualized in a t-SNE embedding with the help of the “ggplot2” R package. A gene set variation analysis (GSVA) was conducted to uncover enriched biological pathways among the high and low G-AUC subgroups. The results were represented in a bar chart, displaying all the significantly different pathways.

### 2.4 Gene set enrichment analysis (GSEA)

This study determined the absolute enrichment fraction of a specified gene set in every sample by applying ssGSEA. To assign glycosylation enrichment values to each participant in the TCGA-UM cohort, we employed ssGSEA. Based on their glycosylation enrichment scores, participants were divided into two groups, high-GSN and low-GSN, for further investigation.

### 2.5 Weighted gene co-expression network analysis (WGCNA)

The systems biological method WGCNA was applied to the gene co-expression network of TCGA-UM (23). The following outlines the steps are taken: exclusion of genes with missing values using the “goodSamplesGenes” function, grouping of tumor samples, deletion of outliers, and establishment of a cut line of 100. The optimal soft threshold for adjacency calculation was determined using graphical methods. An adjacency matrix was generated from the expression matrix to determine the genetic interconnectedness of the network. The topological overlap matrix (TOM) was then constructed from the adjacency matrix. Hierarchical clustering was performed using an average linkage approach and the differences in TOM. The hierarchical clustering tree was dynamically pruned to identify similar modules with high correlation coefficients (r > 0.25). Pearson’s correlation test was applied to examine the relationship between eigengenes and clinical characteristics. The modules containing genes with the most significant correlations to clinical traits, such as glycosylation score, survival status, and survival time were selected for further investigation.

### 2.6 Construction of the risk scoring

A venn diagram analysis was conducted to pinpoint the intersection between the DEGs and the target genes in WGCNA. This was followed by a univariate analysis of the overlapping genes to select those that showed a statistically significant correlation with patients’ OS (P < 0.01). The least absolute shrinkage and selection operator (LASSO) analysis was then employed to narrow down further the list of genes and risk coefficients strongly linked to prognosis, creating a risk model using the “glmnet” package. Based on the coefficients obtained from the LASSO analysis, a risk score was assigned to each UM patient. The patients in the TCGA-UM dataset were divided into two groups, GCNS_low and GCNS_high, using the median risk score as the cutoff. The Kaplan–Meier (K-M) method was utilized to generate prognostic survival curves. The performance of the predictive model was evaluated employing receiver operating characteristic (ROC) curves, with a good performance defined as an area under the curve (AUC) value of > 0.8. The accuracy of the signature in predicting outcomes was demonstrated by using survival analysis and AUC value in an independent dataset (GSE84976). PCA was carried out to reduce dimensionality and visualize the differences between the two risk groups. The same analysis was performed on the GSE84976 cohort.

### 2.7 Assessment of the prognostic model’s independence and validity

A nomogram combining GCNS, age, gender, and the pathological stage was developed to estimate the 1-, 2-, and 3-year OS probability (24). The accuracy of the nomogram was assessed through ROC curves and calibration curves. The net benefit of the nomogram and individual clinical features was also evaluated through decision curve analysis (DCA). Subgroup analysis was performed to determine the prognostic value of the GCNS among subpopulations defined by specific clinical characteristics, including age, gender and clinical stage.

### 2.8 Assessment of the relationship between prognostic models and tumor immunity and its impact on immunotherapy

We analyzed the immune infiltration level of UM patients in the TCGA database using data from the TIMER 2.0 database, which comprises seven evaluation methods. We then conducted a ssGSEA analysis of genes in the prognostic model with the “GSEABase” package to determine immune-related properties. The “estimate” R package facilitated the calculation of the relative proportions of stromal cells, immune cells, tumor cells and their comparison across different GCNS categories. A higher score indicates a greater presence of components in the TME. Furthermore, several immune cell-expressed molecules serve as immunological checkpoints that regulate the level of immune activation and prevent excessive immunological activation (25). We compared the expression levels of both groups of well-known immune checkpoint genes (ICGs) extracted from the literature. To assess their potential in predicting immunotherapy response, tumor immune dysfunction and exclusion (TIDE) was applied. Finally, we evaluated the IMvigor210 cohort to confirm the ability of the GCNS model to predict immunotherapy outcomes.

### 2.9 Mutational landscape and drug sensitivity

From the TCGA database, gene mutation profiles of UM patients were obtained, and the “maftools” software was used to display them. The GCNS and the comprehensive gene mutation files were combined. GCNS_low and GCNS_high groups’ signaling pathways were compared using GSEA, and the essential active pathways in the GCNS_high group were identified. To establish the half-maximal inhibitory concentrations (IC50) of common chemotherapeutic drugs, we also used the R package “pRRophetic,” which allowed us to look into the relationship between the GCNS and drug sensitivity (26). Wilcoxon signed-rank tests compared the IC50 values between the two GCNS groups.

### 2.10 Cell culture and transfection

The Cell Resource Center at Shanghai Life Sciences Institute provided the human uveal melanoma cells (MuM-2B, OCM-1) used in this study. The cells were cultured in DMEM (Dulbecco’s Modified Eagle’s Medium) (Gibco, USA) with 1% penicillin/streptomycin and 10% fetal bovine serum (FBS) (Gibco, USA) in a humid incubator (37°C and 5% CO_2_). Cells were sown in six-well plates and cultured at 37°C to 80% confluence before transfecting. Ribobio created the si-AUP1 and si-NC (control) (Ribobio, Guangzhou, China). Then, following the manufacturer’s instructions, they were transfected into MuM-2B and OCM-1 cells using Lipofectamine 3000 (Invitrogen, Carlsbad, CA, USA). After the transfection had been going on for 48 h, more research was done. AUP1 siRNA sequences were given in **Supplementary Table S1**.

### 2.11 Real time-polymerase chain reaction (RT-PCR)

Using TRIzol reagent (15596018, Thermo, Waltham, MA, USA), total RNA was extracted from MuM-2B and OCM-1 cells, and RNA purity and concentrations were measured using the manufacturer’s recommendations. When creating cDNA using the PrimeScriptTM RT reagent Kit (R232-01, Vazyme, Nanjing, China), the following settings were made: 15 min at 37°C, then 5 s at 85°C, and finally storage at -20°C. The PCR procedure was performed using a 10 μL volume in 40 cycles of 95°C for 10 s and 60°C for 30 s. Three times each operation was carried out. GAPDH was used as a reference standard, and the relative gene expression was analyzed using the 2^-ΔΔCt^ technique. Tsingke Biotech company created specific primers (Tsingke, Beijing, China). In **Supplementary Table S1**, used primers were supported.

### 2.12 Cell proliferation

CCK-8 was used to determine how AUP1 affected the ability of UM cells to proliferate. UM cells were grown in triplicate in 96-well microplates with a cell density of 5,000 per well. Following transfection, the cells were treated at 37°C for 2 h with 10 μL of CCK-8 solution (A311-01, vazyme, Nanjing, China) mixed with 90 μL of complete media in each well at 0, 24, 48, 72, or 96 h. Finally, the absorbance of each well was measured at 450 nm using a microplate reader. The EdU test was used as an additional technique to quantify cell proliferation using the EdU proliferation detection kit (Ribobio, Guangzhou, China). In a nutshell, EdU was applied to MuM-2B and OCM-1 cells (2×10^5^ cells per well) for 2 h before they were stained with DAPI (Thermo Fisher Scientific, USA). A fluorescent microscope (Olympus, Japan) was used to take pictures of the EdU-positive cells, which were then processed in ImageJ.

### 2.13 Transwell migration

The Transwell migration test was used to detect cell migration in a 24-well transwell plate with 8 m-pore membrane filters. In a nutshell, 10% FBS was added to the media in the bottom chamber, and 2×10^5^ cells in serum-free medium were coated on the top chamber. After a 48-hour incubation period, the cells that had migrated to the chamber’s bottom were bathed in 4% methanol for 10 minutes before being stained for 15 min with 0.1% crystal violet (Solarbio, Beijing, China). The images were taken using a microscope’s eyepiece, and the number of migrating cells was counted using ImageJ software.

### 2.14 Wound-healing assay

The wound healing experiment reflects the MuM-2B and OCM-1 cells’ migratory patterns. 80% confluence was obtained by the transfected cells after they had been cultured in a six-well plate and incubated at 37°C. A sterile 200 μL pipette tip left a liner scrape in cell monolayers. The medium was changed to one without serum following two PBS washes to remove cell debris. Under an inverted microscope, the distance that cells traveled into the wound surface was gauged at 0 h and 48 h (Olympus, Japan). Lastly, we examined the wound region using ImageJ software. Data were shown as the rate of relative cell migration.

### 2.15 Animal models

All animal studies were authorized by the Nanjing Medical University Animal Experiment Ethics Committee. Null BALB/c mice that were five weeks old were utilized as the xenotransplantation model. MuM-2B cells that were stably transfected with AUP1 and control cells were implanted into mice’s left and right groins to conduct tumorigenic studies. The tumor volume was calculated every five days. The tumor from the xenograft was removed and weighed 25 days after injection.

### 2.16 Statistical analysis

R software, namely version 4.2.0, was used to conduct our analyses. Student t-tests or one- or two-way ANOVAs with Bonferroni’s multiple comparison *post hoc* tests were used to determine statistical significance in GraphPad Prism 8 (La Jolla, CA, USA). Three times each operation was carried out. The mean and standard deviation (SD) of the data were shown. With a p-value of 0.05, the result was considered statistically significant.

## 3 Results

### 3.1 scRNA profiling of uveal melanoma

**Figure 1:**
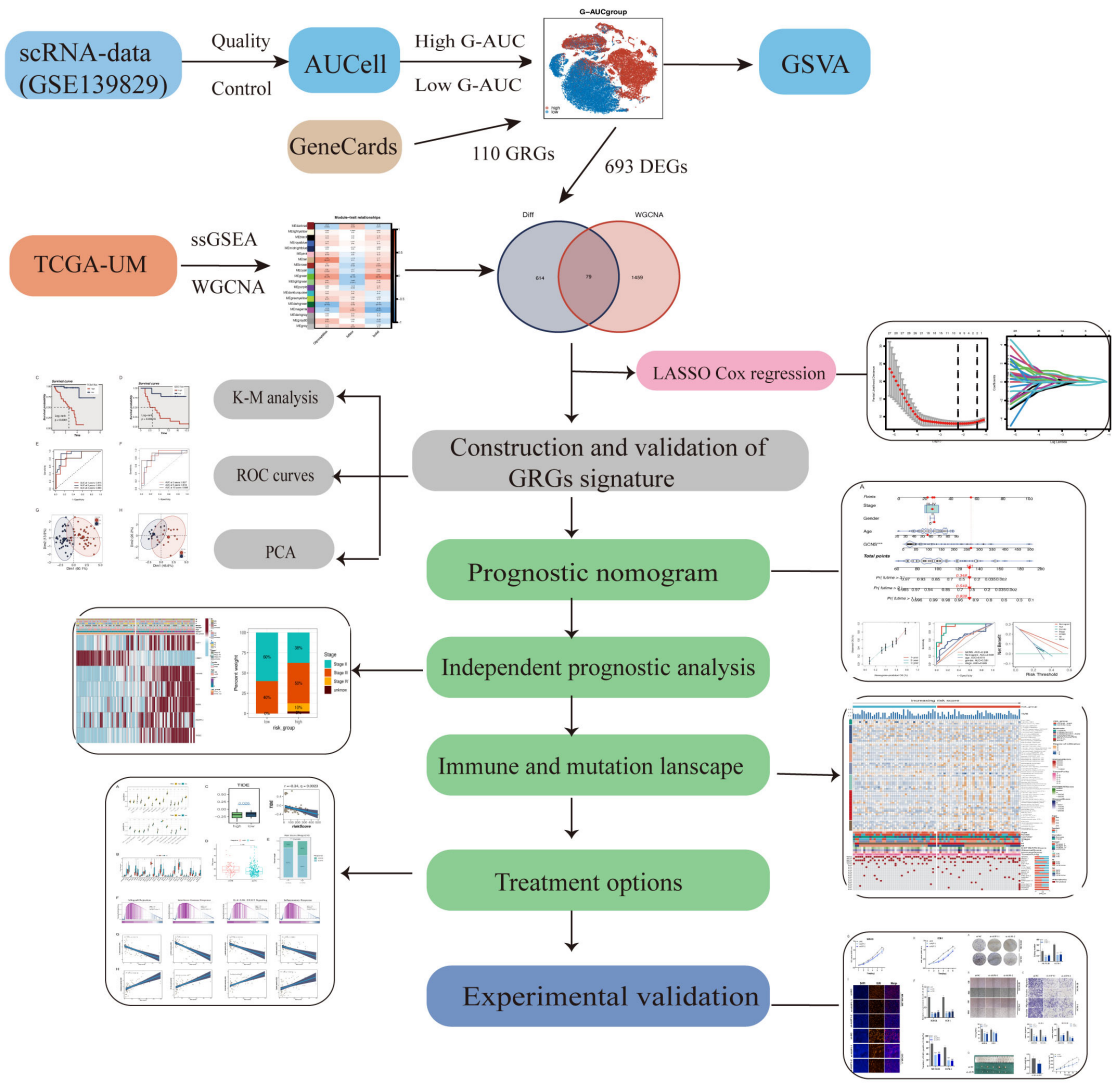
The technical roadmap of this study.

**Figure 2:**
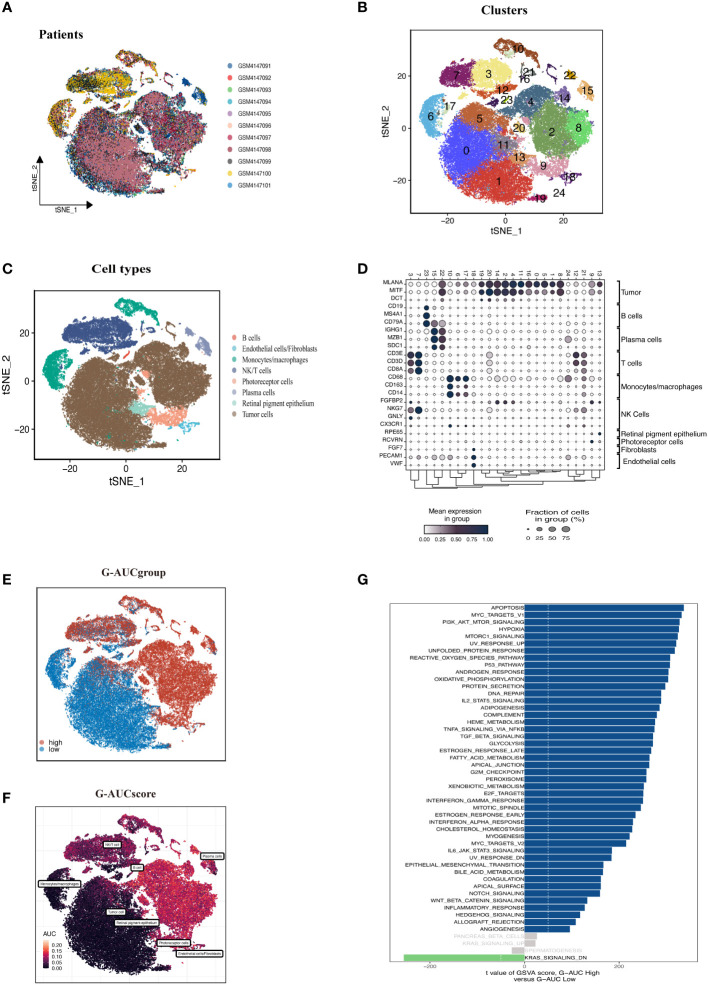
Overview of the single-cell landscape of UM tumor samples of GSE139829. **(A)** The t-SNE plot shows the integration of 11 samples. Cells were evenly distributed among all samples, suggesting no significant batch effects among the UM clusters. **(B)** After quality control and standardization, all cells in 11 samples revealed 24 cell clusters marker with unique colors. **(C)** The cells were annotated into eight categories of cell types according to the composition of the marker genes, namely B cells, endothelial cells, monocytes/macrophages, NK/T cells, photoreceptor cells, plasma cells, retinal pigment epithelium, and tumor cells. **(D)** Dot plot of cell type marker genes. Cell-specific marker genes were selected according to previous studies. The color of the dots represents the average expression, and the size represents the average percentage of cells expressing the desired gene. **(E)** Visualization of the percentage of GRGs in each cell *via* the AUCell package. The cells were divided into high and low groups, namely high G-AUC and low G-AUC subgroups. **(F)** t-SNE plots of the AUC score in all clusters. B cells and plasma cells express more GRGs and exhibit higher AUC values. **(G)** GSVA analysis revealed significant enrichment pathways between the high G-AUC and low G-AUC groups; blue represents the enrichment pathway in the high G-AUC group, and the green represents the pathway involved in the low G-AUC group.

**Figure 1** shows the process used in this investigation. 28,981 cells were deleted after quality screening using the aforementioned standards. The eleven samples included in the investigation had no observable batch effects since the distribution of cells within each piece was pretty uniform (**Figure 2A**). Then, using the t-SNE approach, all cells were divided into 24 more specific clusters depending on all levels of gene expression (**Figure 2B**). We used differential expression analysis to find DEGs—cluster marker genes—for several clusters. These clusters were recognized as known cell lineages using “singleR” package annotation and previously reported marker genes (**Figure 2C**). An image of the expression of cell type-specific marker genes is shown in **Figure 2D**. There are eight kinds of cells, including tumor cells, monocytes/macrophages, and endothelial cells/fibroblasts. We could examine the GRGs expression patterns by measuring each cell line’s GRGs activity using the “AUCell” package (**Figure 2E**). The AUC values were higher in cells that expressed more genes, and in this study, most of these cells were orange-colored B cells and plasma cells (**Figure 2F**). According to the AUC score threshold values, all cells were given an AUC score for the associated GRGs, and they were then split into two groups (high and low G-AUC subgroups). To understand the likely biological processes behind these variations, we conducted differential and functional analyses. According to GSVA data, we discovered apoptosis, MYC targets V1, and the PI3K/AKT/mTOR signaling pathway were particularly prevalent in the high G-AUC subgroups (**Figure 2G**).

### 3.2 WGCNA

**Figure 3:**
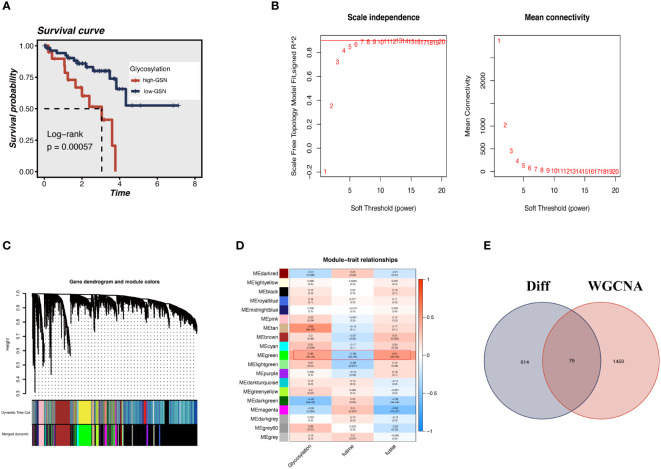
ssGSEA and WGCNA. **(A)** The glycosylation score for each UM patient in the TCGA database was calculated. UM patients in the high-GSN group had worse outcomes (P<0.001), suggesting that glycosylation is a risk factor for UM. **(B)** We applied WGCNA to construct the gene co-expression networks of UM patients. The distribution and trends of scale-free topological model fit, mean connectivity and soft threshold. **(C)** The clustering of genes among different modules by the dynamic tree cut and merged dynamic method. **(D)** Heatmap shows the average correlations among module eigengenes and clinical features. The correlation coefficient and p-value (in parentheses) are shown. The MEgreen module is closely related to glycosylation and survival time, marked with red frames. **(E)** The Venn diagram shows the intersection of the DEGs identified between high G-AUC and low G-AUC groups and MEgreen module genes obtained from WGCNA.

Each TCGA-UM sample received a glycosylation score from ssGSEA, as shown in **Figure 3A**. Patients were split into high-GSN and low-GSN groups depending on the median glycosylation score. The survival analysis discriminated between the high-GSN and low-GSN groups. Glycosylation may be a risk factor for UM since we discovered that patients in the high-GSN group had increased mortality (P < 0.001). WGCNA was utilized to narrow the possible GRGs strongly associated with UM prognosis (**Figure 3B**). 19 non-gray modules were produced with these settings (soft domain value = 7, minimum number of modules = 100, deepSplit = 3, similarity threshold = 0.25) (**Figures 3C, D**). The relationships between phenotypic traits and each module’s expression were evaluated. The DEGs and MEgreen module’s 79 overlapping genes were then chosen to be examined in the subsequent phases (**Figure 3E**).

### 3.3 Establishment of GRGs signature for prognosis prediction

**Figure 4:**
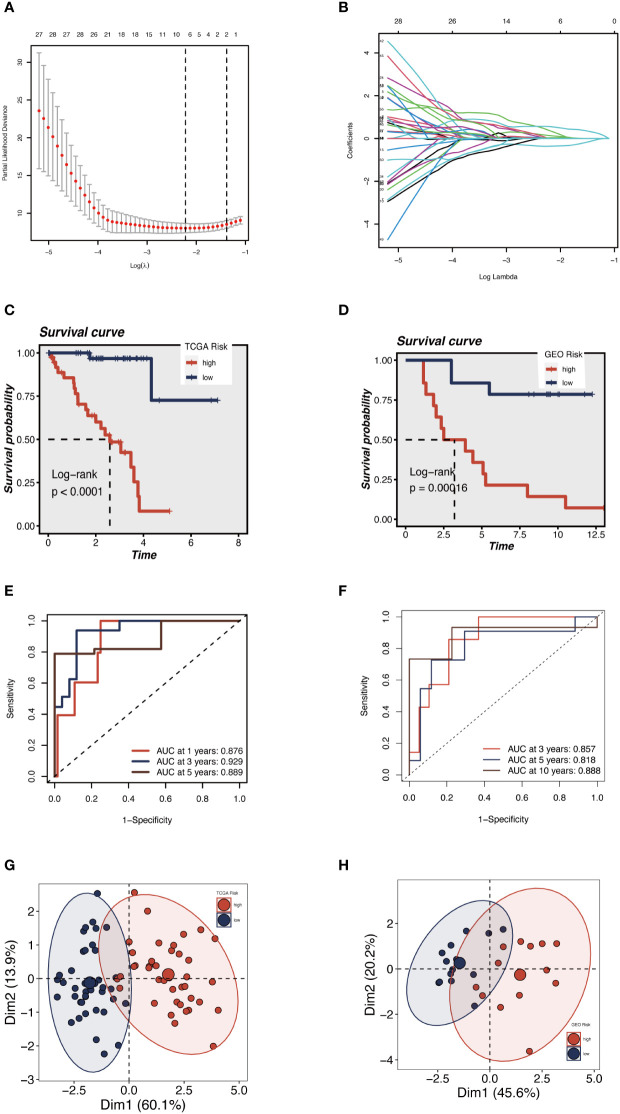
Construction and validation of the 5 GRGs model in TCGA-UM cohort and GSE84976. **(A)** 10-fold cross-validation for tuning parameter selection in the LASSO model. **(B)** The Y-axis shows LASSO coefficients and the X-axis is −log (lambda). Dotted vertical lines represent the minimum and one standard error values of lambda. Differences in OS in different risk subgroups in TCGA-UM cohort **(C)** and GSE84976 cohort **(D)** were assessed using the log-rank test. Compared to low-risk UM patients, a shorter OS is found in high-risk UM patients. **(E)** Time-dependent ROC curve depicting the predictive accuracy of the risk model for OS at 1-, 3- and 5-year in the training set (AUC = 0.876, 0.929, and 0.889, respectively). **(F)** The AUC value of the risk score for predicting 3-, 5- and 10-year survival in the validation cohort (GSE84976) were 0.857, 0.818 and 0.888, respectively. The PCA demonstrates that the model can distinguish patients into GCNS_high and GCNS_low groups well in the training set **(G)** and validation set **(H)**.

We sought to create a GRGs prognostic signature based on the previously mentioned 79 intersected genes to investigate further the connection between GRGs and the prognosis of UM patients. When we initially used the TCGA-UM cohort as our training set for univariate Cox analysis, we discovered 63 genes to be substantially (P < 0.01) linked with the OS of UM patients. Next, the prognostic model was created using LASSO Cox regression analysis (**Figures 4A, B**). Finally, seven GRGs (AUP1, HNMT, PARP8, ARC, ALG5, AKAP13, and ISG20) were filtered out with optimal regularization settings. Patients in the TCGA cohort were divided into GCNS_high and GCNS_low groups based on their median risk ratings. According to K-M analysis, individuals in the GCNS_high group served a lower survival time than those in the GCNS_low group (P < 0.001) (**Figure 4C**). We also assessed the connection between GCNS and OS in GSE84976 to demonstrate the predictive power of GCNS. Using the same technique, we assigned each patient a GCNS and divided them into two groups. The two groups showed a clear difference in survival analyses, with the GCNS_high groups showing a worse prognosis than the GCNS_low groups, which is consistent with earlier findings (**Figure 4D**). The training cohort’s AUC at 1, 3, and 5 years was 0.876, 0.929, and 0.889, respectively, showing that our model was incredibly influential in predicting UM patients’ prognosis (**Figure 4E**). In the validation set, similar outcomes were attained. Additionally, ROC analysis revealed that the AUC of the model value varied between 0.81 and 0.88, demonstrating the outstanding predictive accuracy of our GCNS model (**Figure 4F**). PCA well-distinguished patients in the various GCNS groups, showing that the model can stratify risk subtypes in both the training and validation cohorts (**Figures 4G, H**).

### 3.4 Development and validation of prognostic nomogram

**Figure 5:**
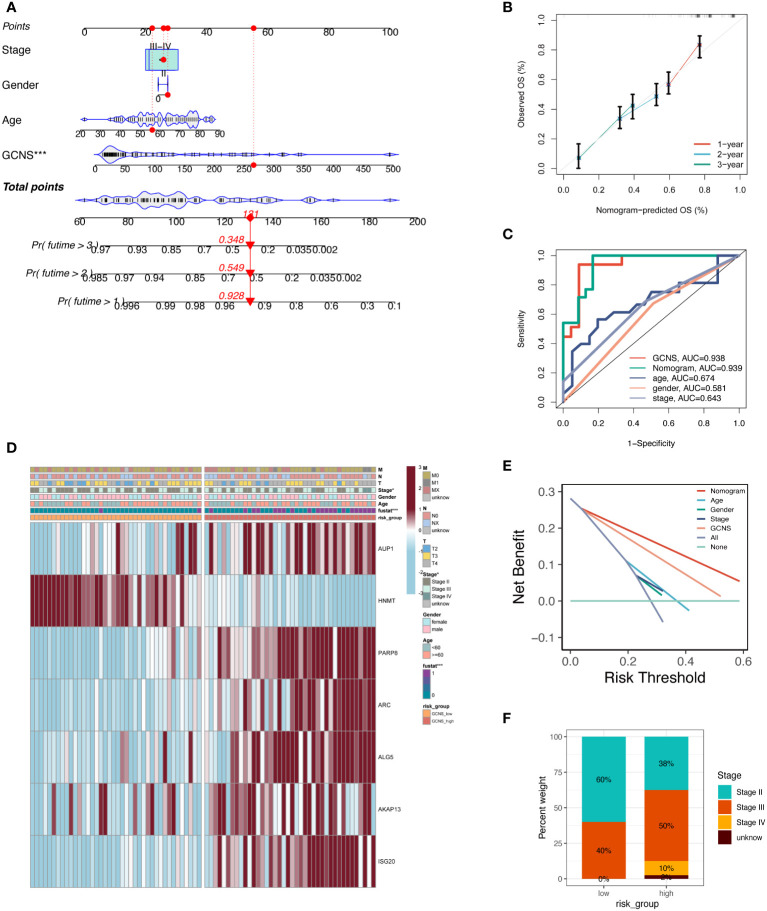
Development and evaluation of prognostic nomogram integrating GCNS and conventional clinical traits. **(A)** A nomogram was generated to evaluate the 1, 2, and 3-year survival rates of UM patients based on the TCGA cohort. The red line shows an example of how to predict the prognosis. **(B)** The calibration curve depicted the consistency between nomogram predicted 1-, 2-, and 3-year survival rates of patients and actual survival rates. **(C)** The AUC value predicted by the nomogram for patient prognosis remains about 0.939, which is significantly higher than other clinical features. **(D)** Differences in clinicopathologic features and expression levels of GRGs between the GCNS_high and GCNS_low subtypes. **(E)** DCA curve was drawn to compare the clinical efficacy of the nomogram based on the threshold probability. The upper lines indicate more net benefit. **(F)** UM stage III and IV patients accounted for the largest proportion in the GCNS_high group and increased significantly compared to the GCNS_low group.

An integrated GCNS and clinical parameters prognostic nomogram was created to forecast the prognosis of UM patients. Clinical results at 1, 2, and 3 years were used to calculate the patients’ survival rates (**Figure 5A**). The calibration plot demonstrated that the GRGs signature offered exact estimates of UM patients’ OS (**Figure 5B**). The nomogram has more extraordinary predictive ability than any clinical trait, as shown by the ROC curve’s AUC of 0.939. (**Figure 5C**). DCA plots showed that adding clinical variables to GCNS might increase the precision of survival prediction (**Figure 5E**). The clinical stage and survival status showed a favorable link to a heatmap of clinical variables and prognostic indicators of GRGs. However, other clinical characteristics did not vary statistically (**Figure 5D**). A percentage bar plot was used to compare the distributions of certain stages among the groups. According to our research, stage II patients make up the majority of patients in the GCNS_low group, whereas stage III patients are in the GCNS_high group (**Figure 5F**). UM patients were divided into subgroups based on unique clinical characteristics, and the GCNS’s ability to predict outcomes was evaluated in each group. Additionally, we saw that patients with GCNS_high consistently had reduced survival chances in all categories, which suggests that the prognostic model applies to all situations.

### 3.5 Tumor microenvironment components

**Figure 6:**
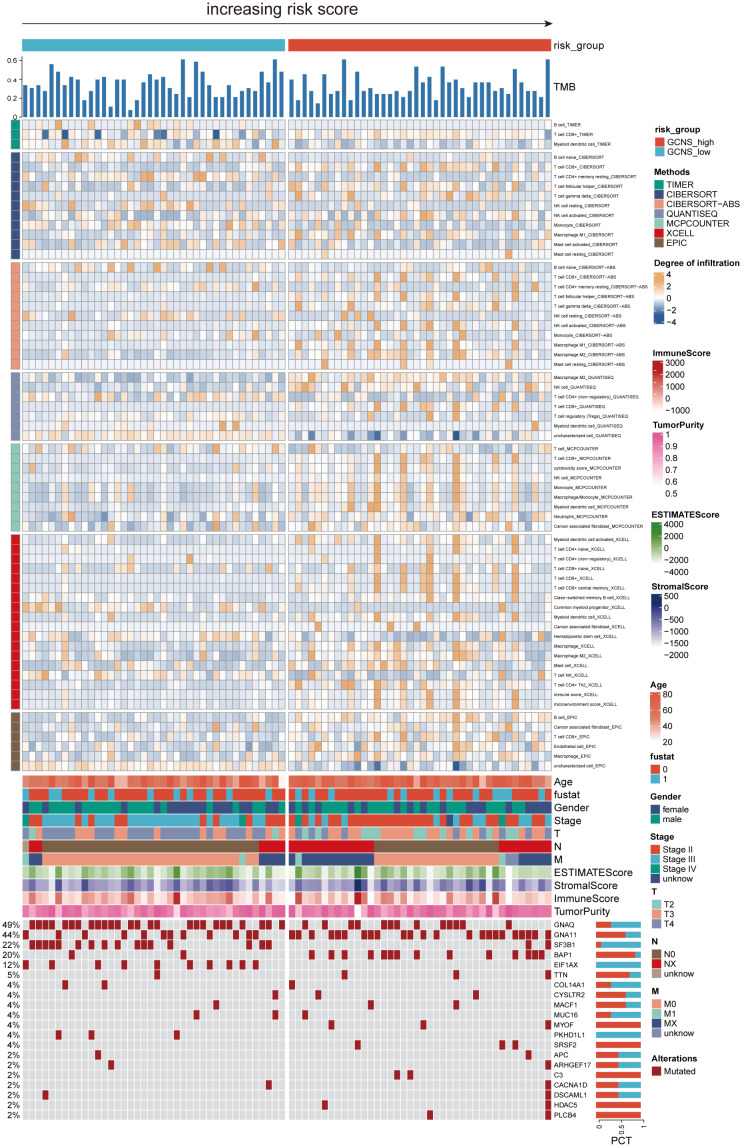
The landscape of immune and stromal cell infiltrations in the GCNS_high and GCNS_low groups. The heatmap shows the normalized scores of immune and stromal cell infiltrations according to the evidence from the TIMER database. The Wilcoxon Test compared the two groups’ statistical differences in immune cell infiltration. For the GCNS_high group, blue-gray represents cells with lower infiltration, while yellow represents cells with higher infiltration. The GCNS_high group tended to have higher levels of immune cell infiltration. The TMB calculated by package “maftools” was also displayed. Patient’s clinical features and gene mutation patterns were also illustrated as an annotation.

Given the significant differences in OS amongst GCNS subgroups, we anticipated that the immune milieu would be critical in tumor formation and clinical outcomes. Therefore, we looked for distinctive immunological characteristics in the TCGA-UM patients. **Figure 6** illustrates how patients with GCNS_high exhibited higher immune cell infiltration, including M2 macrophage cells and B cells. According to the estimating methodology, patients with high GCNS had significantly higher immune scores, stromal scores, and estimate scores (stromal score plus immune score) than those with low GCNS. According to the data, a relationship exists between GCNS and the amount of immune cell infiltration and TME components. Various rates of disease onset and immunotherapeutic efficacy may result from different immune infiltration levels. We assessed somatic mutation profiles of UM patients based on GCNS in light of the intrinsic link between genetic mutation and tailored treatment for cancer patients. The top three mutant genes were GNAQ, GNA11, and SF3B1, as shown in **Figure 6**. Combining the mutational gene distributions with GCNS, we found the most prevalent mutation in GCNS_low patients in GNAQ, whereas the most frequent mutation in GCNS_high patients was in GNA11. This discrepancy may help to explain why these groups respond to immunotherapy so differently.

### 3.6 Immunotherapy and chemotherapy response prediction

**Figure 7:**
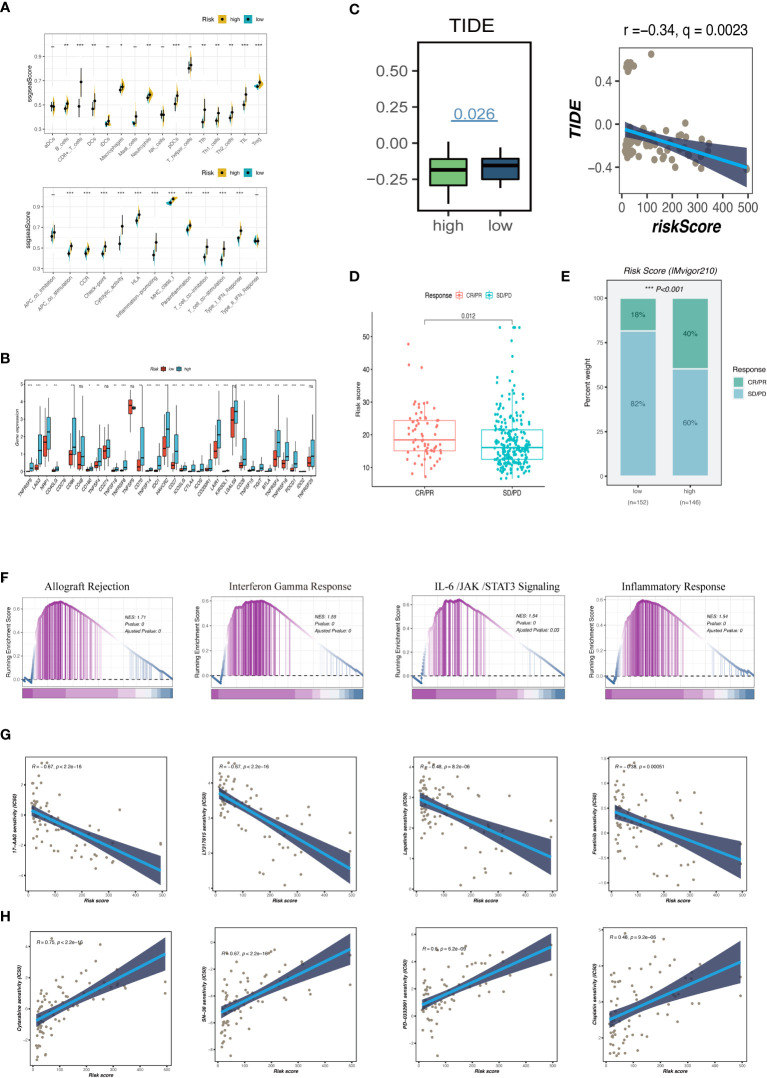
Analysis of immune infiltration, immunotherapy and enrichment pathways. **(A)** The differences of tumor-infiltrating of 16 cell types and the score of immune pathways between the GCNS groups by ssGSEA. Between-group differences were evaluated using the Wilcoxon test. The black dots represent the median values, and asterisks indicate significance. **(B)** The differences in expression levels of ICGs between the GCNS_high and GCNS_low subtypes. The lines inside the boxes represent the median values, and the lines outside indicate the 95% confidence interval. **(C)** Prediction of response to immunotherapy. TIDE score was low in the GCNS_high group. Pearson correlation analysis showed a negative correlation between GCNS and TIDE. **(D)** Comparing risk scores in groups with different anti-PD-L1 treatment response statuses in the IMvigor210 cohort. R represents complete response (CR)/partial response (PR); NR represents progressive disease (PD)/stable disease (SD). **(E)** The comparison of the proportion of non-responders and responders to anti-PD-L1 immunotherapy between the two GCNS groups in the IMvigor210 cohort. **(F)** GSEA showed that allograft rejection, IL-6/JAK/STAT3 signaling, and inflammatory response pathways related to immune regulation were activated in the GCNS _high group. **(G, H)** Comparison of the IC50 values of chemotherapy agents in the two GCNS groups. The predicted IC50 values of 17-AAG, LY317615, lapatinib and foretinib were generally lower in the GCNS_high group, whereas cytarabine, SN-38, PD-0332991 and cisplatin had a lower IC50 in the GCNS_low group. *P < 0.05; **P < 0.01; ***P < 0.001.

To support these findings, we used ssGSEA to compare the immune cell makeup of two GCNS groups (**Figure 7A**). Those with high GCNS had significantly more partial innate immune cells (like macrophages and DC cells) and adaptive immunity cells (like B cells and T cells) than patients with low GCNS. The GCNS_high subgroup also had higher enrichment scores for functions created linked to the immune system. These results imply that immunological glycosylation-related characterization is more prevalent in the GCNS_high group. We looked at the possibility of this prognostic model to forecast UM patients’ responses to ICI therapy. We examined the relationship between the TCGA-UM cohort’s GCNS and the most common immunotherapeutic targets. Nearly all ICGs showed noticeably greater expression in the GCNS_high group, including PDCD1 (PD-1), CD274 (PD-L1), CTLA4 and LAG3 (**Figure 7B**). As shown in **Figure 7C**, the immunotherapy responses in the GCNS groups were contrasted. One of the key characteristics of cancer that depends on the tumor’s ability to survive in the human body is immune system evasion. TIDE is a valuable biomarker for predicting the response to immunotherapy in patients with diverse malignancies, particularly those treated with ICI. This evaluation measures the immune escape capability of tumors (27). In patients taking anti-PD-1 and anti-CTLA-4 therapy, the poorer the ICI response, the higher the tumor TIDE score. We found that patients with high GCNS had significantly lower TIDE scores and a negative association between GCNS and TIDE values (P < 0.001, | r | > 1).

The risk of a tumor immune escape increased as the TIDE value increased. However, the effectiveness of immunotherapy has decreased. We could infer from this that those with high GCNS are better candidates for immunotherapy. Subsequently, we evaluated the ability of our model to predict the efficacy of immunotherapy using the IMvigor210 cohort to confirm the validity of this discovery. The number of patients receiving anti-PD-1 therapy who saw an objective and partial response increased as the risk score rose (**Figures 7D, E**). According to our findings, patients in the GCNS_high group had a higher chance of benefiting from immunotherapy. The GCNS may be a biomarker to pinpoint the right patient population for immunotherapy.

To examine the differences in route enrichment between the GCNS_high and GCNS_low groups, GSEA was used. We discovered that allograft rejection, IL-6/JAK/STAT3 signaling, and the inflammatory response were enriched in the GCNS_high group, suggesting that GCNS_high patients are intimately connected to immune regulation-related and inflammatory pathways (**Figure 7F**). In order to broaden the practical application of the prognostic model, we forecast how susceptible patients in the GCNS_ high and GCNS_low groups would be to chemotherapeutic drugs. Lapatinib, foretinib, LY317615 and 17-AAG all had lower IC50 values in the GCNS_high group, indicating that GCNS_high patients respond better to these medications (**Figure 7G**). There was a strikingly negative correlation between drug sensitivity and GCNS for cytarabine, SN-38, PD-0332991 and cisplatin, suggesting that these drugs may be more effective in treating GCNS_low people identified by our model (**Figure 7H**).

### 3.7 AUP1 promoted the proliferation, migration, and invasion abilities of UM cells

**Figure 8:**
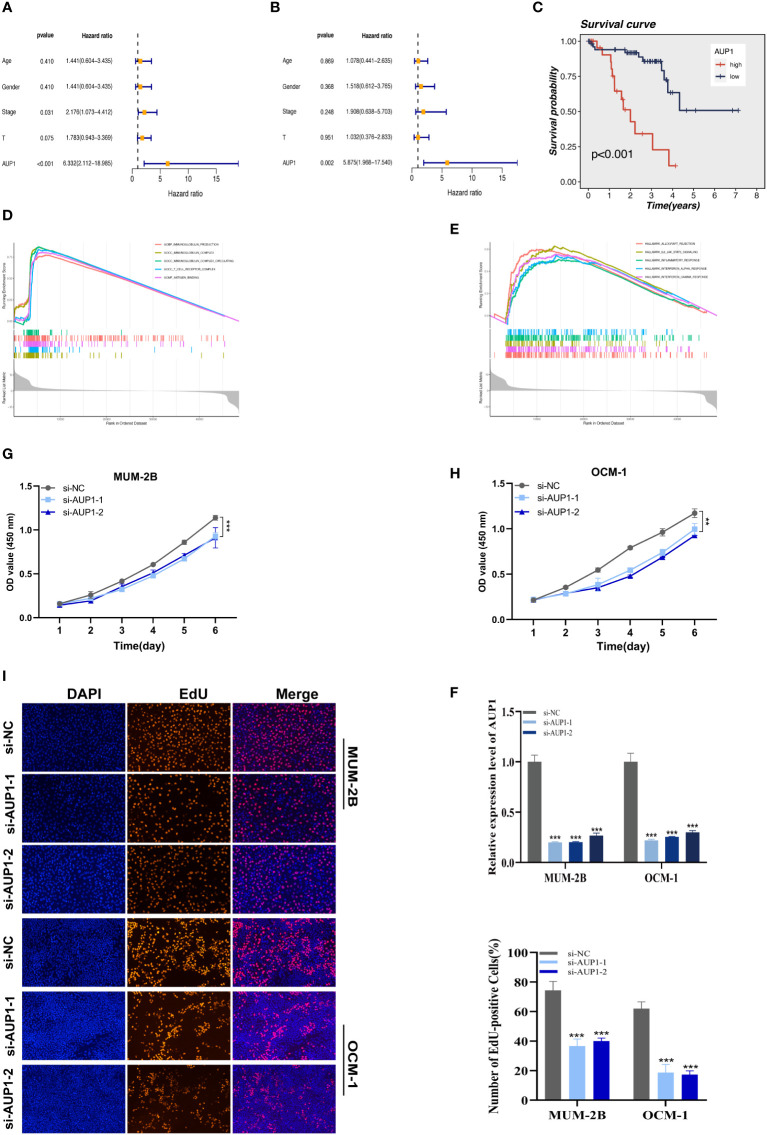
The results of univariate **(A)** and multivariate **(B)** Cox regression indicated that the AUP1 was an independent prognostic factor for OS in UM patients. **(C)** Survival analysis of AUP1 in TCGA database. High expression of AUP1 is associated with a poor prognosis of UM. **(D)**. GO analysis of AUP1 high expression group. **(E)** GSEA enrichment plots represented enriched biological pathways in high AUP1 groups. **(F)** The role of the critical gene AUP1 in uveal melanoma cell lines was verified in vitro. Both siRNAs significantly down-regulated AUP1 expression in MuM-2B and OCM-1 cell lines. **(G, H)** The CCK-8 assay showing the proliferation ability of MuM-2B and OCM-1 cells decreased significantly after silencing AUP1. **(I)** EdU staining assay indicated that downregulation of AUP1 expression repressed MuM-2B and OCM-1 cell proliferation. **P < 0.01; ***P < 0.001.

**Figure 9:**
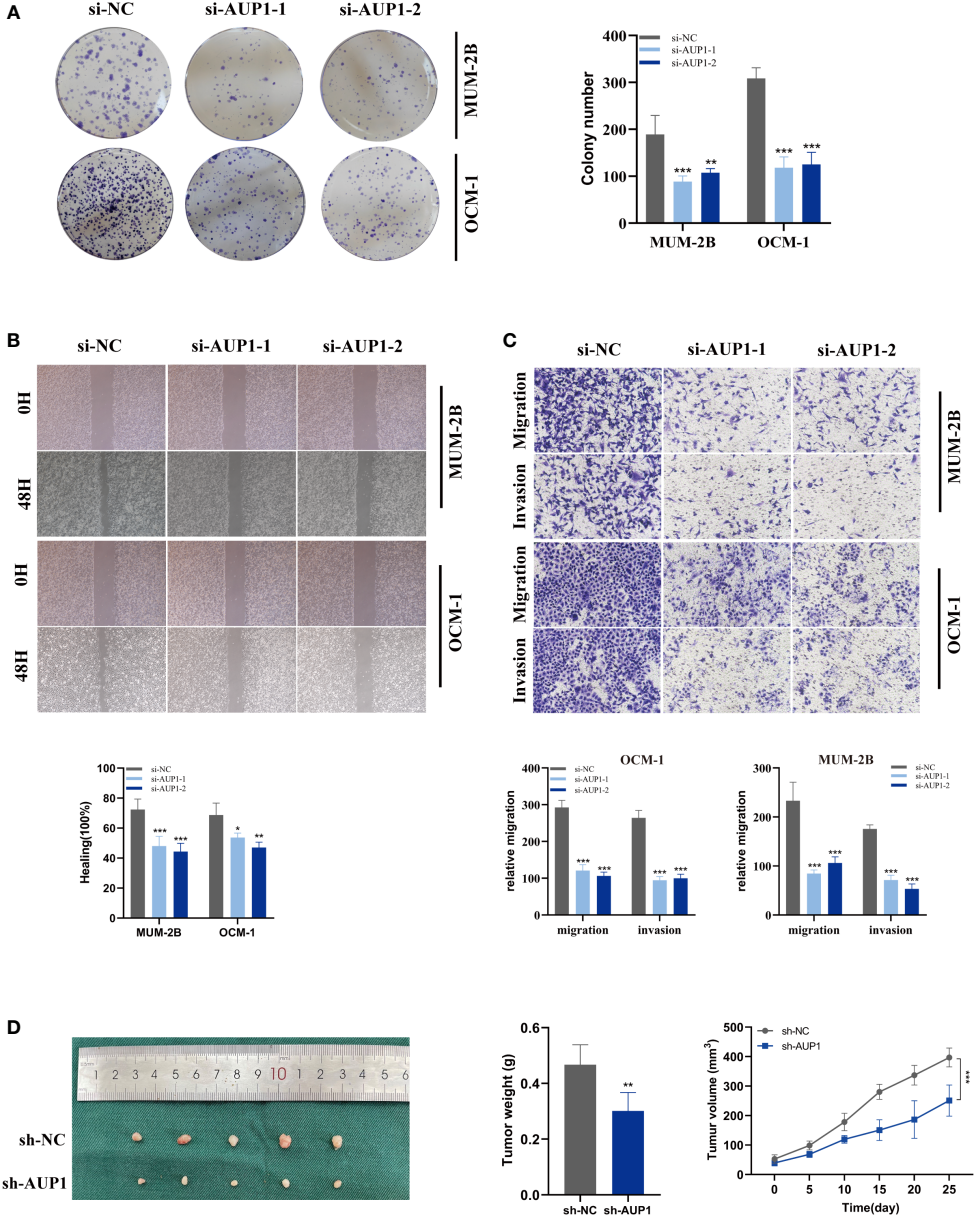
Related experiments for AUP1. **(A)** Colony formation assays revealed that the ability of the MuM-2B and OCM-1 cell lines to produce colonies was considerably reduced following AUP1 knockdown. **(B, C)** AUP1 knockdown dramatically reduced the migration and invasion capacity of MuM-2B and OCM-1 cell lines in the wound healing and transwell experiment. **(D)** Experiments using naked mice. AUP1 knockdown decreased tumor growth, and tumor volume and weight were lower in the knockdown group than in the control group. All tests were performed in two UM cell lines (MuM-2B and OCM-1) to verify the accuracy and reproducibility of the results. *P < 0.05; **P < 0.01; ***P < 0.001.

Using univariate and multivariate Cox analysis, the predictive value of AUP1 was contrasted with that of other clinicopathological factors. Forest plots showed that AUP1 had the highest HR among the clinical features, suggesting that AUP1 constituted a separate risk factor for predicting the prognosis of UM patients (**Figures 8A, B**). Patients with high AUP1 expression had a significantly poorer prognosis than those with low AUP1 expression (**Figure 8C**). In light of these results, AUP1 was chosen for further *in vitro* testing to confirm its role in UM. GO analysis showed that high AUP1 expression groups were mainly focused on immunoglobulin production, immunoglobulin complex and antigen binding, suggesting the expression of AUP1 was related to immune regulation and metabolism (**Figure 8D**). According to GSEA, high-AUP1 groups were significantly enriched in allograft rejection, IL6/JAK/STATA3 signaling and inflammatory response signaling pathways (**Figure 8E**). The AUP1 knockdown system was created in MuM-2B and OCM-1 cells (**Figure 8F**). The CCK-8 and EdU assays revealed that AUP1 silencing decreased the proliferation rate of UM cells (**Figures 8G–I**). Clonal formation experiments simultaneously showed that the MuM-2B and OCM-1 cell lines’ capacity to form colonies was significantly diminished (**Figure 9A**). Additionally, the transwell test and wound healing experiment revealed a lower tendency for UM cell migration and invasiveness following the reduction of AUP1 compared those transfected with si-NC (**Figures 9B, C**). Comparing AUP1 knockdown to controls, tumor growth, size, and weight were all reduced (**Figure 9D**). These results suggest that AUP1 was involved in regulating pro-oncogenic processes in UM.

## 4 Discussion

About 50% of patients with UM die from metastatic UM, the leading cause of mortality in this population (28). Due to the unique characteristics of the ocular anatomy, systemic medication administration in UM patients is frequently suboptimal (29). Because of this, researchers in the UM area are motivated to provide more accurate approaches for identifying and managing metastatic illness. A more profound comprehension of the complex ecology of UM is necessary to define the therapy goals for UM patients.

Over 60 years ago (30), the first report of glycosylation variations connected to oncogenic transformation appeared. The disruption of crucial functions within cancer cells and the TME by various types of glycoconjugates is thought to contribute to the growth and spread of cancer (31). Several physiopathological processes may be controlled through glycosylation, which incorporates a number of enzymes, organelles, and other elements to produce post-translational alterations linked to carbohydrates (11). Due to glycosylation’s susceptibility, even minor pathogenic alterations or metabolic stress can cause it to malfunction, creating abnormal glycochains and glycoproteins (14). Understanding the causes and consequences of glycosylation changes linked to tumor illness will offer priceless insights into tumor development (11). The complete picture of glycosylation in UM could be more intricate. Therefore, more studies must be done.

Single-cell sequencing technology has made it possible to examine the diverse tumor environment and extract gene expression from UM tumor cells at the individual cell level, essential for identifying the treatment targets for UM patients (32, 33). In this study, using bulk RNA-seq and scRNA data, we built a GCNS model for UM patients and examined the expression patterns of the GRGs. We first identified numerous cell subpopulations inside UM and discovered that the activity of GRGs differed throughout cell lineages, focusing on increased glycosylation activity in B cells and plasma cells. The high G-AUC subgroup was strongly enriched in apoptosis, MYC targets V1, and PI3K/AKT/mTOR signaling pathways, all of which deserve in-depth research in the future, according to GSVA algorithm.

Next, using LASSO algorithm on the TCGA dataset, a prognostic model based on seven OS-related GRGs was created and validated using GSE84976. UM patients were classified into GCNS_high and GCNS_low groups, with those in the GCNS_high group displaying a worse prognosis independent of clinical parameters. We investigated the underlying mechanism after the prognostic signature showed a robust predictive capacity for prognosis in both the training and validation groups. As anticipated, there were differences in the levels of immune infiltration, TMB and immunotherapy response between the GCNS_high and GCNS_low groups, which may cause the heterogeneity of UM tumors.

A growing number of studies have shown that TME is intimately connected to carcinogenesis and can, to some degree, predict tumor prognosis and the effectiveness of immunotherapy. The immune system is suppressed, and lymphatic circulation is restricted in the eye, which eventually causes the CD8+ T cell population to decline (34, 35). High levels of M2-type macrophages and CD8^+^ T cells are found in the UM immune milieu in the GCNS_high group. CD8^+^ T cells emerge as a critical player in the tumor immunosurveillance system, indicating a bad prognosis for UM patients. The two G subunit genes, GNAQ and GNA11, which are mutually exclusive, frequently have to activate mutations in UM (36). Notably, the GNAQ mutation was more widespread in the GCNS_low group, whereas the GNA11 mutation was more prevalent in the GCNS­_high group. Mutations in GNAQ and GNA11 activate pathways that might serve as a foundation for using MEK or Akt inhibitors in clinical settings (37, 38), thus providing a reference for optimizing targeted therapy in UM patients.

Tumor immunotherapy has quickly advanced, and it is now becoming clear that its primary goals are to stop tumor cells from evading the immune system, boost the body’s immunological reaction to tumor cells, and cause immune-received tumor cells to die (39, 40). James P Allison and Tasuku Honjo disclosed numerous immunological checkpoints’ immunosuppressive modes and created ICI based on this to block PD-L1/PD-1/CTLA4 (41, 42). ICI in clinical trials have significantly improved cancer treatment in some cancer types, including melanoma. Contrary to previous study’s findings, we concluded that immunotherapy would be successful for those in the GCNS_high group using the TIDE algorithm and data from the IMvigor210 cohort. To obtain exact and individualized treatment, we propose giving each UM patient a risk score based on a prognostic model, ascertaining whether they fall into the GCNS_high or GCNS_low group, and treating UM patients in the GCNS_high group with immunotherapy. Rather than PD-1 and CTLA4, the critical sign of failure in UM is the suppressive immunological checkpoint of LAG3 (43). This partially explains why anti-PD-1 and anti-CTLA4 treatments are ineffective. LAG3 is highly expressed in tumor-infiltrating lymphocytes in UM, as Triozzi et al. discovered in 2014 (44). There are several clinical studies evaluating the therapeutic effectiveness of LAG3 inhibitors in treating various malignancies, one of which (NCT02519322) uses relatlimab to treat advanced UM (45).

Clarifying the function of modeling genes in controlling glycosylation in UM is necessary to offer new treatment options for malignancy. Our analysis of seven modeled genes showed that AUP1 had the greatest HR value. A subsequent survival study showed elevated AUP1 expression levels were significantly associated with a poorer clinical outcome in UM patients. Of note, suppressing AUP1 expression significantly inhibited the proliferation and invasiveness of UM cells. Based on the studies, AUP1 is a prospective clinical biomarker for UM. Meisler first recognized and defined AUP1, which contains 410 amino acids and is found on human chromosome 2p13 in a conserved linkage region (46). AUP1 has an “ancient conserved area” in proteins from unrelated organisms (47). Due to its age and high level of sequence conservation, the protein encoded by AUP1 is essential for cell biology (48). However, the function of AUP1 in UM has yet to be determined. The AUP1 high and low expression groups were compared using GSEA to determine which biochemical pathways were significantly enriched in either group. The results of GSEA identified 5 AUP1-associated enriched pathways, and the IL-6/JAK/STAT3 signaling pathway was part of the activated signaling pathway. To our knowledge, the IL-6/JAK/STAT3 signaling pathway is aberrantly hyperactivated in individuals with chronic inflammatory diseases, hematopoietic malignancies and solid tumors (49). Several cell types within the TME release IL-6, activating JAK/STAT3 signaling in both tumor cells and immune cells infiltrating the tumor, promoting tumor-cell proliferation, survival, invasiveness and metastasis (49). Consequently, we speculated that AUP1 is involved in the IL6/JAK/STAT3 signaling pathway to drive the proliferation, invasion and migration of UM cells. However, the crosstalk and mechanism of the above bioinformatics prediction need verification with well-designed experiments.

Despite the favorable results, the research nevertheless contained several flaws. Since UM had a significant degree of heterogeneity and our signature was built and validated using cohorts in relatively small sample sizes, it is crucial to confirm the GCNS propensity for prognostication in a big multicenter cohort before applying the model in clinical practice. Additionally, we were only concerned with how AUP1 silencing affected UM cell proliferation, invasion, and migration. The description of the potential relationship between the expression of AUP1 and the prognosis for UM. More research is still needed to determine how AUP1 contributes to the development and progression of UM tumors through glycosylation alteration. The predictive biomarker potential of our risk model creates fresh treatment options for UM.

## Data availability statement

The original contributions presented in the study are included in the article/**Supplementary Material**. Further inquiries can be directed to the corresponding authors.

## Ethics statement

The animal study was reviewed and approved by The Nanjing Medical University Animal Experiment Ethics Committee.

## Author contributions

JL, PZ, and FY contributed conception and design of the study; KJ, SS, and ZX collected the data; JL, PZ, and KJ performed the statistical analysis; JL, PZ, FY, and KJ wrote the first draft of the manuscript; JL, GY, and JT revised the manuscript; JT provided technical and material support; GY and JT gave the final approval of the version to be submitted. All authors contributed to the article and approved the submitted version.
