# Supplementary material for: Integrating single-cell analysis and machine learning to create glycosylation-based gene signature for prognostic prediction of uveal melanoma
Source: Front Endocrinol (Lausanne). 2023 Mar 23;14:1163046. doi: 10.3389/fendo.2023.1163046 (PMC10076776; doi:10.3389/fendo.2023.1163046)
Supplement: Supplementary file 4 [file Table_1.docx]

| **Oligonucleotides** | **Nucleotide sequence (5'-3')** |
| --- | --- |
| **siRNA** |  |
| SiRNA-NC | GCUUCGCGCCGUAGUCUUA |
| Si SYAP1-1 | GCTGCTAAGCAAGATGAGATT |
| Si SYAP1-2 | CCTTCGATGCCTGTAACCTAA |
| **Primer** |  |
| GAPDH | GGCCTCCAAGGAGTAAGACC (forward) |
|  | AGGGGAGATTCAGTGTGGTG (reverse) |
| SYAP1 | GCCTTCGATGCCTGTAACCT (forward) |
|  | TTCCAGTACGGCTGTCTCCT (reverse) |
|  |  |

**Table S1. Oligonucleotides used in research**
